# Extracellular Vesicle miR-122-5p as a Prognostic Biomarker in Pediatric Classical Hodgkin Lymphoma

**DOI:** 10.3390/ijms252413243

**Published:** 2024-12-10

**Authors:** Rebekka J. S. Salzmann, Anna Garbin, Enrico Gaffo, Caterina Elia, Gaia Martire, Stefania Bortoluzzi, Annalisa Tondo, Paola Muggeo, Alessandra Sala, Marco Pizzi, Marta Pillon, Elisa Carraro, Egesta Lopci, Valli de Re, Maurizio Mascarin, Lara Mussolin

**Affiliations:** 1 Istituto di Ricerca Pediatrica “Città della Speranza”, 35128 Padua, Italy; rebekkajohannasabine.salzmann@studenti.unipd.it (R.J.S.S.); anna.garbin.13@gmail.com (A.G.); gaia.martire@studenti.unipd.it (G.M.); 2 Maternal and Child Health Department Pediatric Hematology, Oncology and Stem Cell Transplant Center, University of Padua, 35128 Padua, Italy; marta.pillon@unipd.it (M.P.); elisa.carraro@unipd.it (E.C.); 3 Department of Molecular Medicine, University of Padua, 35128 Padua, Italy; enrico.gaffo@unipd.it (E.G.); stefania.bortoluzzi@unipd.it (S.B.); 4 AYA Oncology Unit, Department of Radiation Oncology, Centro di Riferimento Oncologico IRCCS, 33081 Aviano, Italy; eliacaterina@libero.it (C.E.); mascarin@cro.it (M.M.); 5 Department of Pediatric Oncology and Haematology, Meyer Children’s Hospital IRCCS, 50139 Florence, Italy; annalisa.tondo@meyer.it; 6 Department of Pediatric Oncology and Hematology, AOU Policlinico Consorziale di Bari-Ospedale Giovanni XXIII, 70124 Bari, Italy; paola.muggeo@gmail.com; 7 Pediatric Hematology Oncology Unit, Fondazione IRCCS San Gerardo dei Tintori, 20900 Monza, Italy; alessandra.sala@irccs-sangerardo.it; 8 Pathology Unit, Department of Medicine—DIMED, University of Padua, 35128 Padua, Italy; marcopizzi2002@yahoo.it; 9 Nuclear Medicine Unit, IRCCS—Humanitas Research Hospital, Rozzano, 20089 Milano, Italy; egesta.lopci@cancercenter.humanitas.it; 10 Immunopatologia e Biomarcatori Oncologici, CRO Aviano, National Cancer Institute, Istituto di Ricovero e Cura a Carattere Scientifico, IRCCS, 33081 Aviano, Italy; vdere@cro.it

**Keywords:** Hodgkin lymphoma, extracellular vesicles, miRNA, relapse, biomarkers, liquid biopsy, inflammation, T-cells, immune escape, diagnostics

## Abstract

Currently, risk stratification for pediatric Hodgkin lymphoma is based on clinical factors such as stage, bulk, and systemic symptoms. Novel minimally invasive biomarkers could enhance both prognosis and treatment strategies. Therefore, the plasma extracellular vesicles’ microRNA profile was characterized by small RNA sequencing in 36 classical Hodgkin lymphoma cases and these findings were confirmed in an extended cohort of 86 patients by RT-qPCR. It was found that the levels of miR-122-5p at diagnosis were significantly higher *(p*-value: 0.0002) in patients who relapsed compared to patients in remission. The 5-year event-free survival of cases with high and low levels of miR-122-5p was 65 ± 7% and 93 ± 4%, respectively. MiR-122-5p levels were significantly associated with clinical events in both univariate *(p*-value: 0.0009) and multivariate *(p*-value: 0.0037) analysis (hazard ratio 5.8). Target prediction analysis suggests an involvement in the polarization of immune cells. The phenotypic characterization of peripheral blood mononuclear cells in 12 patients showed significantly increased levels of CD4+ T-cells in cases with high miR-122-5p levels as compared to low levels *(p*-value: 0.048). Moreover, CCL17 (TARC) and IL-6 plasma levels at diagnosis were significantly higher as compared to healthy donors *(p*-value: ≤0.0001). MiR-122-5p could complement current prognostic assays to identify patients at high risk of relapse.

## 1. Introduction

Classical Hodgkin lymphoma (cHL) accounts for approximately 5–6% of all childhood cancers and is by far the most common malignancy in adolescents [1]. Histologically, cHL is characterized by a small number of neoplastic cells, referred to as Hodgkin and Reed–Sternberg cells, scattered throughout a rich and heterogeneous inflammatory background consisting of plasma cells, histiocytes, eosinophils, and neutrophils [2]. Intense chemotherapy, autologous stem cell transplants (ASCT) [3,4], and (more recently) the anti-CD30 antibody–drug conjugate brentuximab vedotin and the PD-1 inhibitors nivolumab and pembrolizumab are regarded as front-line treatment in cHL [5,6], leading to 5-year event-free survival (EFS) rates as high as 85% [7]. Patient outcome is still unsatisfactory for patients with relapse (REL) or who are not eligible for ASCT [4,8]. As such, the early prediction of REL could help develop patient-tailored treatments to control disease progression and to improve disease outcome. Despite this, a uniform risk stratification for pediatric cHL is lacking, although several clinical parameters have been associated with poorer prognosis (i.e., the presence of B symptoms, mediastinal and/or peripheral lymph node bulky lesions, the presence of extranodal disease, a high number of involved nodal stations, Ann Arbor stage, high serum markers for inflammation, male gender, and poor response to initial chemotherapy) [9].

MicroRNAs (miRNAs) play key biological roles as tumor suppressors or oncogenes, and plasmatic circulating miRNAs were described as promising disease biomarkers in adult cHL [10]. Therefore, we hypothesized that they could yield comparable prognostic potential in pediatric patients. Blood liquid biopsies can be obtained easily, in relatively large amounts, yielding an abundance of clinically relevant information. Among peripheral blood biomarkers, small extracellular vesicles (sEVs) are present at high levels. Due to their biogenesis through the endosomal pathway, they reflect closely the status of their secreting cells, containing parts of the membrane and cytoplasm together with all the soluble contents such as proteins, lipids, and nucleic acids [11]. Activated cells, especially tumor cells, produce significantly more sEVs than normal cells due to the activation of proto-oncogenes and contribute to the remodeling of the tumor microenvironment and tumor progression [12,13]. Therefore, we decided to investigate the small RNA (sRNA) content of plasma circulating sEV to identify new disease biomarkers for diagnosis, monitoring, and prognostic stratification.

Thus, the primary objective of this study is to identify novel liquid biopsy markers to minimally invasively predict pediatric cHL outcomes. sRNA sequencing analysis and RT-qPCR revealed an upregulation of miR-122-5p in plasma sEVs of pediatric cHL at the day of diagnosis (DIA) in REL compared to patients achieving complete remission (CR). In univariate analysis, it was found that there is a significant correlation of 5-year EFS with Ann Arbor stage, Erythrosedimentation rate (ESR) bulky disease, treatment level, and miR-122-5p levels at DIA. In multivariate analysis, the prognostic potential of miR-122-5p was confirmed.

In a prior study from our group [14], it was reported that miR-122-5p levels in circulating sEVs at the DIA were elevated in pediatric anaplastic lymphoma kinase-positive (ALK+) anaplastic large cell lymphoma (ALCL) as compared to healthy donors (HDs) and that this miRNA was barely detectable in ALK+ ALCL-involved lymph nodes, in reactive lymph nodes, and extranodal skin biopsies. Indeed, miR-122-5p is expressed in a variety of neoplasms, including breast, lung, ovarian, esophageal, colorectal, liver, and urothelial carcinomas as well as in various hematological malignancies. In such tumors, miR-122-5p acts as both an oncogene and a tumor suppressor [15,16,17,18]. MiR-122-5p deregulation and aberrant expression in carcinogenesis and tumor development also suggests a potential role for this miRNA as a diagnostic and/or prognostic marker.

## 2. Results

### 2.1. Relapsed Patients Display a Distinct Small Extracellular Vesicle microRNA Cargo Compared to Patients in Complete Remission

**Figure 1:**
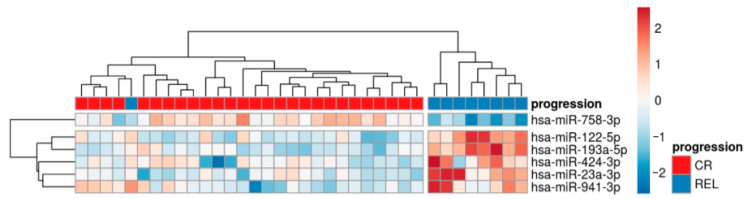
cHL patients that later relapsed (REL) have a specific microRNA (miRNA) signature in plasma-derived small extracellular vesicles (EVs) at diagnosis. Heatmap representing differentially abundant miRNAs at diagnosis between REL and complete remission (CR) cHL patients with miR-122-5p is the most upregulated miRNA in the cHL cohort compared between REL and CR. Total RNA from plasma sEVs of 36 cHL patients (CR = 25, REL = 11) was sequenced.

sRNA sequencing on total RNA isolated from the plasma sEVs of thirty-six pediatric cHL patients and seven pediatric HDs was performed. The principal component analysis revealed a slightly different miRNA cargo between HDs and cHL (Supplementary Figure S1A). In total, 25 miRNAs were found to be more and 21 to be less enriched in cHL compared to HDs (Supplementary Figure S1B). Then, the sRNA profiles of cHL cases based on clinical factors, including PET 2, low intermediate risk and high risk, B symptoms, extranodal lesions (E lesions), Ann Arbor stage, bulky disease, and disease progression (Supplementary Figure S2) were compared. A comparative analysis of disease progression revealed the deregulation of sEV miRNAs in REL patients compared to patients in CR (Figure 1). The downregulated miRNA in REL patients, miR-758-3p has functional roles as tumor suppressors [19] while the upregulated miR-122-5p and miR-424-3p have been linked to tumor-supporting functions [14,20].

**Figure 2:**
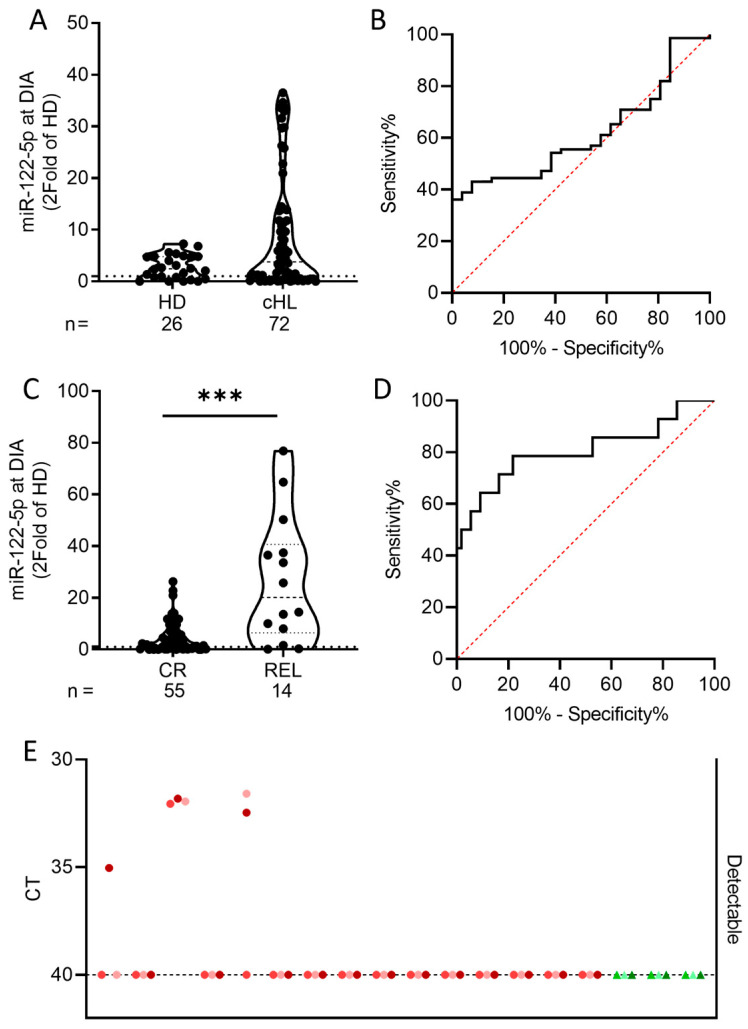
RT-qPCR analysis of miR-122-5p in 86 pediatric cHL plasma samples, 27 pediatric healthy donor (HD) plasma samples, 15 primary tumor biopsies from pediatric cHL patients and 3 cHL cell lines. (**A**,**B**) Outliers (1 HD and 14 cHL) were removed by the ROUT (Q = 1%) method. At DIA miR-122-5p expression is not significantly different in plasma small extracellular vesicles (sEVs) of cHL patients compared to HDs. Unpaired student’s two-tailed t-test analysis of cHL vs. HD: not significant (*p*-value: 0.0836, ROC = 0.6149, 95% CI: 0.502–0.7277). (**C**,**D**) Outliers (14 CR and 3 REL) were removed by the ROUT (Q = 1%) method. At DIA miR-122-5p expression is significantly different in plasma sEVs of CR compared to relapsed (REL) patients. Unpaired student’s two-tailed t-test analysis of CR vs. REL: significant (*p*-value: 0.0002, ROC = 0.8065, 95% CI: 0.6495–0.9635). (**E**) In the cHL cell lines KM-H2, L-428, and L-540 (green) and primary tumor biopsies (red) at DIA the miR-122-5p is barely or not detectable. Data are expressed in triplicates as cycle threshold (CT) with CT 40 being the limit of detection.

The miR-122-5p levels in plasma sEVs by RT-qPCR were assessed in an extended cohort of 86 pediatric cHL patients and 27 HDs and outliers were removed by the ROUT (Q = 1%) method. Before outlier removal, miR-122-5p levels in HD and cHL patients differed significantly (Mann–Whitney test, *p*-value: 0.0098, **) (Supplementary Figure S3). After outlier removal (1 HD and 14 cHL), a trend of increased levels in cHL as compared to HDs *(p*-value: 0.0840, Figure 2A,B) and a significant increase in miR-122-5p in REL as compared to CR patients *(p*-value: 0.0002, ***, Figure 2C,D) was observed. Next, the miR-122-5p levels in fifteen primary tumor biopsies of cHL patients and in three HL cell lines (KM-H2, L-428 and L-540) were measured. MiR-122-5p was undetectable in almost all primary tumor samples and in all cell line-derived sEVs. These findings are in line with a previous study on ALK+ ALCL from our group, showing that miR-122-5p was undetectable in tumor samples, the skin, and reactive lymph nodes of ALK+ ALCL patients, with the notable exception of liver biopsies [14] (Figure 2E). This observation is supported by miR-122-5p being a liver tissue-specific miRNA [21], suggesting that it is not tumor tissue derived but most likely originates from the liver tissue. Of note, liver involvement at presentation is very rare in cHL [22]. Interestingly, the miR-122-5p levels at follow-up (FUP) compared to DIA were increased significantly (*p*-value: ≤0.0001, ****, Supplementary Table S1).

### 2.2. MiR-122-5p Levels at Diagnosis Predict 5-Year Event-Free Survival

**Figure 3:**
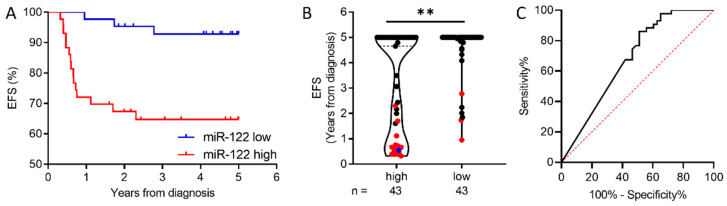
miR-122-5p has prognostic potential based on the stratification into miR-122 low and miR-122 high according to the median expression values. (**A**) The 5-year event-free survival (EFS) according to patient stratification into miR-122 low and miR-122 high. The miR-122 low and miR-122 high cohorts contain 43 patients, respectively. In the miR-122 low cohort, 3 patients experienced an event with a 5-year EFS of 93%. The miR-122 high cohort experienced 15 events with one death with a 5-year EFS of 65%. (**B**) Dot plot representing EFS with a 5-year cut-off of 86 cHL patients (CR = 68, REL = 18) stratified into miR-122 high and miR-122 low in plasma small extracellular vesicles (sEVs) at DIA. Events are marked in red; death is marked in blue. The 5-year event-free survival was significantly different between the miR-122 high and miR-122 low groups (*p*-value: 0.0016, **). (**C**) ROC curves of miR-122 low and miR-122 high as a control (AUC 0.679, *p*-value 0.0043, 95% CI: 0.5649–0.7931).

The stratification of 86 cHL patients into 43 miR-122 low and 43 miR-122 high cohorts based on the median expression levels as the fold of HDs predicts a 93 + 4% 5-year EFS for patients with miR-122 low and 65 + 7% with miR-122 high (*p*-value: 0.0016, **, AUC 0.679) (Figure 3A–C). An extended statistical analysis of 5-year EFS with all available clinical parameters, including B symptoms, mediastinal and/or peripheral lymph node bulk, extranodal disease, number of nodal sites, Ann Arbor stage, serum markers for inflammation, gender, and response to initial chemotherapy, and the miR-122-5p stratification at DIA was performed. In the univariate analysis, a significant correlation of 5-year EFS and the stage at presentation (*p*-value: 0.0014), the ESR (*p*-value: 0.026), bulky disease (*p*-value: 0.007), treatment level (*p*-value: 0.0037), and the miR-122-5p stratification at DIA (*p*-value: 0.0009) was found. In multivariate analysis, however, only the miR-122-5p stratification at DIA remained significant (*p*-value: 0.0037) (Table 1). Additionally, a positive correlation between miR-122-5p levels and the body mass index (BMI) (Supplementary Table S1) was found.

### 2.3. cHL Patients Show a Different Peripheral Immune Environment Based on Small Extracellular Vesicle Packed miR-122-5p Abundance

To elucidate the mechanisms of miR-122-5p, the MiRTarBase [23] was entered and all the functional miRNA–target interactions from the list of validated miR-122-5p targets were selected. Based on these targets, a REACTOME [24] analysis was conducted and the signaling by receptor tyrosine kinases was found to be the most enriched with targets of miR-122-5p. Among them there is SOCS1, which acts as a negative regulator of the JAK/STAT signaling pathway which regulates the polarization of CD4+ T-cells [25,26]. 

**Figure 4:**
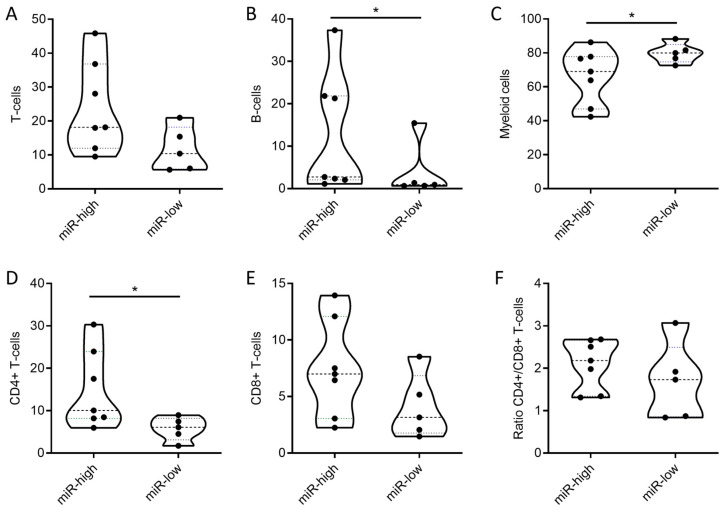
Flow cytometric characterization of peripheral blood mononuclear cells (PBMCs) from 12 pediatric cHL patients stratified into miR-122 high and miR-122 low based on the median expression value in plasmatic small extracellular vesicles (sEVs). Data were analyzed with GraphPad Prism (Version 8.0.2., GraphPad Software, Boston, MA, USA) using a Mann–Whitney test (confidence interval 95%, * *p* ≤ 0.05). (**A**) Percentage of T-cells characterized by CD3 + CD45+, (*p*-value: 0.1061). (**B**) Percentage of B-cells characterized by CD19 + CD45+, (*p*-value: 0.048). (**C**) Percentage of myeloid cells characterized by CD33 + CD45+, (*p*-value: 0.1061). (**D**) Percentage of CD4+ T-cells characterized by CD3 + CD45 + CD4+, (*p*-value: 0.048). (**E**) Percentage of CD8+ T-cells characterized by CD3 + CD45 + CD8+, (*p*-value: 0.202). (**F**) Ratio of CD4+/CD8+ T-cells, (*p*-value: 0.3434).

Next, miR-122-5p levels were measured and peripheral blood mononuclear cells (PBMCs) were characterized in 12 paired cHL plasma and peripheral blood samples obtained at diagnosis. Patients stratified as miR-122 high showed a specific peripheral immune environment characterized by significantly higher levels of B-cells (Figure 4B) and CD4+ T-cells (Figure 4D). The population of myeloid cells, however, was significantly reduced in miR-122 high patients (Figure 4C).

### 2.4. Plasma Levels of CCL17 (TARC) and IL-6 Are Elevated in cHL Compared to Healthy Donors at the Time of Diagnosis

**Figure 5:**
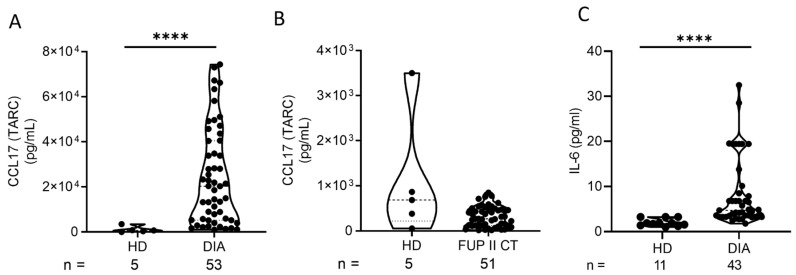
Plasma CCL17 (TARC) and IL-6 levels measured by ELISA. Data were analyzed with GraphPad Prism (Version 8.0.2., GraphPad Software, Boston, MA, USA) using a Mann–Whitney test (confidence interval 95%, **** *p* ≤ 0.0001). (**A**) CCL17 plasma levels at day of diagnosis (DIA) in cHL are significantly higher than in healthy donors (HDs) (*p*-value: ≤0.0001, ****). (**B**) CCL17 plasma levels at follow-up in HDs and cHL are not significantly different (*p*-value: 0.1253, ns). (**C**) IL-6 plasma levels at DIA in cHL are significantly higher than in HDs (*p*-value: ≤0.0001, ****).

Following our observation of increased CD4+ T-cells in miR-122 high patients, the circulating plasma levels of the chemokine CCL17 (TARC) and the cytokine IL-6 were measured as both are known to be part of the immune–tumor interaction and contribute to the cancer-promoting effects of inflammation and immune modulation. Moreover, CCL17 (TARC) was reported to be a highly sensitive diagnostic marker in pediatric cHL [27]. CCL17 (TARC) levels in 53 cHL patients at DIA and 5 HDs were measured, and a significant increase in CCL17 (TARC) (*p*-value: ≤0.0001) was found (Figure 5A). At FUP, 51 cHL patients and 5 HDs were measured. However, TARC levels have normalized and do not differ significantly from HDs (Figure 5B). IL-6 plasma levels were measured in the plasma of 43 cHL patients and 11 HDs and were also significantly elevated (*p*-value: ≤0.0001) at DIA compared to HDs (Figure 5C).

## 3. Discussion

In conclusion, the major novel finding of this study is the identification of miR-122-5p as a strong prognostic biomarker in pediatric cHL. Significantly (*p*-value: 0.0002) elevated levels of miR-122-5p in plasma small extracellular vesicles at DIA were associated with an increased risk of REL and inferior 5-year event-free survival. Remarkably, in multivariate analysis, miR-122-5p stratification remained the only significant (*p*-value: 0.0037) predictor of outcomes when accounting for other clinical risk factors such as Ann Arbor stage, ESR, bulky disease, and treatment level. The stratification of patients into miR-122-5p high- and low-level subgroups based on the median expression value demonstrated a clear predictive value, with high levels of miR-122-5p being associating with a significantly higher risk of REL, while lower levels were linked to a better 5-year event-free outcome. Our findings show that miR-122-5p could serve as a minimally invasive, robust, and early predictor of REL risk and thus could complement current clinical prognostic stratification strategies.

Despite the increase in miR-122-5p levels observed at FUP as compared to DIA, there is no significant difference between REL and CR patients at FUP. We hypothesize that the miR-122-5p increase at FUP may be related to chemotherapy-induced liver stress, as it is predominantly expressed in the liver [17]. This transient increase suggests that miR-122-5p is not only a marker of REL but may also reflect broader systemic stress responses. The fact that miR-122-5p was barely detectable in cHL primary tumor samples and cell lines supports the hypothesis that it is released from non-tumor tissues—possibly the liver—like in pediatric ALK+ ALCL [14]. This could hint toward a mechanism in which the tumor progression is driven not by the tumor cells themselves. Furthermore, the positive correlation between miR-122-5p levels and the body mass index (BMI) (Supplementary Table S1) could indicate that miR-122-5p may also serve as a marker for metabolic status in cHL patients.

In addition to its prognostic significance, miR-122-5p appears to have a functional role in promoting REL through its interaction with the SOCS1/JAK/STAT pathway. Target prediction analysis suggests that miR-122-5p suppresses SOCS1, a negative key regulator of the JAK/STAT signaling cascade [28]. However, in cases where miR-122-5p is elevated, SOCS1 suppression may lead to a constant activation of the JAK/STAT pathway, which is implicated in the pathogenesis of various cancers, including cHL, as it promotes cellular survival, proliferation, immune evasion, and inflammation [29,30,31,32,33,34]. The dysregulation of this signaling axis, driven by high miR-122-5p levels might contribute to REL, making this pathway an important focus for further investigations.

A unique characteristic of cHL is the formation of CD4+ T-cell rosettes, surrounding and protecting the tumor cells, aiding in immune evasion [35,36,37,38]. Our findings in a small cohort of 12 patients revealed that pediatric cHL patients with high levels of miR-122-5p exhibited significantly increased numbers of circulating CD4+ T-cells. The correlation between elevated miR-122-5p levels and increased CD4+ T-cell populations suggests that miR-122-5p might play a role in the recruitment of these T-cells, promoting immune escape and REL in cHL patients.

Moreover, our study found significantly elevated levels of the chemokine CCL17 (TARC) and the cytokine IL-6 in cHL patients at DIA, both of which are known to play crucial roles in modulating the tumor microenvironment [39,40]. CCL17 (TARC) is highly secreted by Reed–Sternberg cells [41] and is involved in recruiting CD4 + T-cells to the tumor site where they suppress local immune responses by inhibiting the function of cytotoxic CD8+ T-cells [42]. Similarly, elevated IL-6 levels in cHL patients at DIA suggest a significant role in cHL. IL-6 is known as a central mediator of inflammatory response and a critical activator of the JAK/STAT pathway by the IL-6 classical or trans-signaling pathway [43]. Elevated IL-6 levels contribute to a pro-tumorigenic microenvironment, promoting the differentiation of CD4+ T-cells [44]. Thus, both CCL17 and IL-6 not only serve as markers of immune dysregulation in cHL but also represent potential diagnostic factors that could be used to monitor disease progression.

We must highlight that we primarily report miR-122-5p as a new prognostic marker. An extended cohort for the PBMC characterization and additional markers for the flow cytometric analysis of the PBMCs would give deeper insights into the disease mechanisms. Moreover, the link between CCL17 (TARC), IL-6, and the miR-122-5p levels should be explored. Functional and in vitro experiments are crucial to understand the mechanism of action of this miRNA and could open new therapeutic strategies for high-risk pediatric cHL patients by, e.g., repurposing the anti-miR-122-5p drug Miravirsen, which was being used to treat hepatitis C virus (HCV) infections [18,45]. It was well tolerated and showed prolonged anti-viral activity [46]. Due to the rise in small-molecule treatments for HCV in this context, its application was discontinued but it could be a potential candidate for drug repurposing. However, since miR-122-5p dysregulation impacts key immune pathways and promotes immune evasion, modulating its expression, alongside inhibiting the JAK/STAT pathway, might help to restore immune balance within the tumor microenvironment and improve the effectiveness of current treatment strategies.

## 4. Materials and Methods

### 4.1. Patients and Case Selection

Eighty-six patients were enrolled in the EuroNet-PHL C2 trial, currently adopted for pediatric cHL treatment by AIEOP. The clinical characteristics of pediatric cHL patients are described in Table 2. Peripheral blood samples in sodium citrate (5 mL) were collected before treatment initiation and/or after 2 OEPA cycles and processed in the laboratory within 24 h. Briefly, the samples were centrifuged at 820× *g* for 10 min and supernatants were carefully removed and then re-centrifuged at 2500× *g* for 10 min to avoid contamination by platelets. Plasma aliquots were stored in the Pediatric Oncology Biobank (BBOP) at −80 °C until used. The study was approved by the ethics committee or by the internal review board of each participating institution, and informed consent was obtained from parents or legal guardians before patient enrolment.

### 4.2. Total Extracellular Vesicle RNA Isolation and Quantitative Real-Time PCR

EVs were isolated from 0.5 mL plasma obtained at DIA using a 0.22 µm syringe filter and further processed using the exoRNeasy midi kit (#77144, Qiagen, Hilden, Germany). In brief, this kit uses membrane affinity spin columns to capture EVs from various biofluids and subsequently employs spin columns to isolate RNA from the EVs. Total RNA from cell lysates was isolated using a total RNA Purification Kit (Norgen Biotek Corp., Thorold, ON, Canada). Reverse transcription was performed using the TaqMan™ Advanced miRNA cDNA Synthesis Kit (ThermoFisher Scientific, Waltham, MA, USA) and quantitative PCR (qPCR) of hsa-miR-122-5p (UGGAGUGUGACAAUGGUGUUUG; assay ID 477855_mir, ThermoFisher Scientific, Waltham, MA, USA) was performed in triplicates using TaqMan™ Fast Advanced Master Mix (ThermoFisher Scientific) and TaqMan™ Advanced miRNA Assays. Hsa-miR-26a-5p (UUCAAGUAAUCCAGGAUAGGCU; assay ID: 477995_mir, ThermoFisher Scientific, Waltham, MA, USA) was used as the reference miRNA to normalize sEVs samples [47,48].

### 4.3. Nanoparticle Tracking Analysis

Nanoparticle tracking analysis was conducted on the Nanosight NS300 instrument (Malvern Panalytical, Malvern, UK). The instrument was equipped with a 488 nm laser, a high sensitivity sCMOS camera, and a syringe pump. The plasma sEV samples were mixed and subsequently diluted at 1:1000 in 0.22 µm filtered 1 × PBS. A standard operational procedure of 3 videos of 60 sec per measurement, syringe pump speed of 30 s and a total of 1500 frames were used. The instrument control and data analysis were performed using the NanoSight software (Malvern Panalytical, Malvern, UK, v. 3.1).

### 4.4. Small RNA Sequencing and Bioinformatics Analysis

Small RNA sequencing libraries were prepared as previously reported [14]. Libraries were prepared using the NEBNext Multiplex Small RNA Library Prep Kit for Illumina (New England Biolabs, Ipswich, MA, USA) and sequenced single end on an Illumina HiSeq 4000 platform (Illumina, San Diego, CA, USA) with an average sequencing depth of 15 million reads per sample. Small RNAs were identified and quantified with miR&moRe2 v0.2.375, as previously reported [49]. DESeq2 [47] v1.24.0 was used to normalize read count data and test for differential expression [50]. A Benjamini–Hochberg adjusted *p*-value ≤ 0.05 was considered statistically significant. The sva v3.26.0 package was applied to remove batch effects.

### 4.5. Peripheral Blood Mononuclear Cell Isolation and Flow Cytometric Analysis

Whole peripheral blood samples of 12 cHL patients in sodium citrate (5 mL) were collected before treatment initiation and processed in the laboratory within 24 h. PBMCs were isolated by using the Ficoll-Paque gradient method according to the manufacturer’s instructions. The cells were supplemented with DMSO and stored at −80 °C until further use. The antibodies were used according to the manufacturer’s recommendations in a total volume of 100 µL 1 × PBS. Per staining panel (Table 3), 0.5 × 10^6^ PBMCs, respectively, were stained for 20 min on ice and in darkness. The cells were washed in 1 mL of 1 × PBS and suspended in 500 µL of 1 × PBS. Flow cytometry was performed using the CytoFLEX (Beckman Coulter, Brea, CA, USA) and the instrument control was carried out by using the software Kaluza (Beckman Coulter, Brea, CA, USA, v. 2.2.1). Flow cytometric analysis was performed using FlowJo (Version 10.4, BD Biosciences, Franklin Lakes, NJ, USA).

An optical gating strategy was followed by setting the first gate in FSC-A/SSC-A on the lymphocytes, and then single cells were selected in SSC-A/SSC-H. In the single lymphocytes gate, T-cells were gated in CD3/SSC. Then CD8+ T cytotoxic cells were gated in CD3/CD8 and CD4+ T helper cells in CD3 + CD4+. The naive T-cells were gated in the CD4+ T helper cell gate using the CD4 + CD45RA+. B-cells were gated in CD3/CD19.

### 4.6. Enzyme-Linked Immunosorbent Assay

CCL17 (TARC) and IL-6 were measured using the kits (ThermoFisher Scientific™, Invitrogen™, MA, USA, #EHCCL17, diluted 1:10–1:100, human plasma, and Abcam plc, Cambridge, UK, #ab46042, undiluted, human plasma) according to the manufacturer’s instructions. Briefly, samples were immobilized on respective capture antibodies and labeled with a biotinylated detection antibody. After washing, the streptavidin-HRP complex was added and incubated at RT. The wells were washed again and TMB was added and incubated in darkness. The colorimetric reaction was stopped, and the plate was measured on a Perkin Elmer Victor 3 1420-012 Multilabel Counter Microplate Reader at 450 nm.

### 4.7. Statistical Analysis

MiRNA expression levels were analyzed using the comparative Delta CT method with miR-26a-5p as the endogenous control, and data were expressed as a fold of HD. The statistical analysis was performed using GraphPad Prism (Version 8.0.2, GraphPad Software, Boston, MA, USA) with ROUT (Q = 1%) for outlier detection, followed by Mann–Whitney tests (* *p* ≤ 0.05, ** *p* ≤ 0.01, *** *p* ≤ 0.001) and ROC curve plots (confidence interval 95%, poor: 0.6 ≤ ROC ≤ 0.7, acceptable: 0.7 ≤ ROC ≤ 0.8, excellent: 0.8 ≤ ROC ≤ 0.9), or a log-rank Mantel–Cox survival curve analysis.

## 5. Conclusions

Here, we described, for the first time, the miRNA cargo of sEVs in pediatric cHL patients. Noteworthy, all REL patients displayed high levels of miR-122-5p at DIA. In multivariate analysis, the negative prognostic power of high miR-122-5p could be considered to identify high-risk patients that could be treated with an intensification of current therapies.

## Figures and Tables

**Table 1:** Univariate and multivariate analysis of clinical parameters and the miR-122-5p expression levels using miR-26a-5p as endogenous control and expressed as the fold of healthy donors (HDs). **ESR:** erythrosedimentation rate. **EFS:** event-free survival. **SE:** standard error. **HR:** hazard ratio.

				Univariate			Multivariate	
Characteristics		Patients (n)	Events	5-Year EFS %	SE%	*p*-Value	*p*-Value	HR (95% CI)
Ann Arbor stage	1-2-3	63	8	87	4	0.0014	ns	
	4	23	10	56	10			
ESR (≤30 mm/h)	No	23	1	96	4	0.026	ns	
	Yes	63	17	73	6			
Bulky disease (>10 cm)	No	48	5	89	5	0.007	ns	
	Yes	38	13	66	8			
Treatment level	1 + 2	45	4	91	4	0.0037	ns	
	3	41	14	66	7			
miR-122-5p at diagnosis	High	43	15	65	7	0.0009	0.0037	5.8 (1.8–19.9)
	Low	43	3	93	4			

**Table 2:** Patient clinical data. NS: nodular sclerosis. ERA: early response assessment. LRA: late response assessment. AR: adequate response. IR: inadequate response. NA: not applicable. PD: progressive disease.

Characteristics		Number of Patients
Sex	Female	47
	Male	39
Median age	≤15 years	42
	>15 years	44
Histology *	NS	62
	Other	20
ERA	AR	55
	IR	31
LRA	AR	21
	IR	4
	NA	56
	PD	5

* Data not available for all patients.

**Table 3:** Staining panels of peripheral blood mononuclear cells for flow cytometric analysis. The antibodies listed were employed for cell surface full stains. The dilutions were chosen according to the manufacturer’s recommendations.

Antibody/Isotype *	Fluorochrome	Clone	REF#
**Panel 1**			
CD3/IgG1 Mouse	PC7	UCHT1	6607100
CD4/IgG1 Mouse	FITC	SFCI12T4D11	345768
CD8/IgG1 Mouse	PE	B9.11	A07757
CD45/IgG1 Mouse	ECD	J33	A07784
**Panel 2**			
CD3/IgG1 Mouse	APC-AF750	UCHT1	A94680
CD19/IgG1 Mouse	PC7	J3-119	IM3628
CD33/IgG1 Mouse	PC5	D3HL60.251	IM2647
CD45/IgG1 Mouse	ECD	J33	A07784

* All antibodies were purchased from Beckman Coulter, CA, USA.

## Data Availability

Data is contained within the article and Supplementary Materials.

## Supplementary Materials

The following supporting information can be downloaded at: https://www.mdpi.com/article/10.3390/ijms252413243/s1.
