# Supplementary material for: Extracellular Vesicle miR-122-5p as a Prognostic Biomarker in Pediatric Classical Hodgkin Lymphoma
Source: Int J Mol Sci. 2024 Dec 10;25(24):13243. doi: 10.3390/ijms252413243 (PMC11678363; doi:10.3390/ijms252413243)
Supplement: Supplementary file 1 [file ijms-25-13243-s001.zip › ijms-3314642-supplementary.pdf]

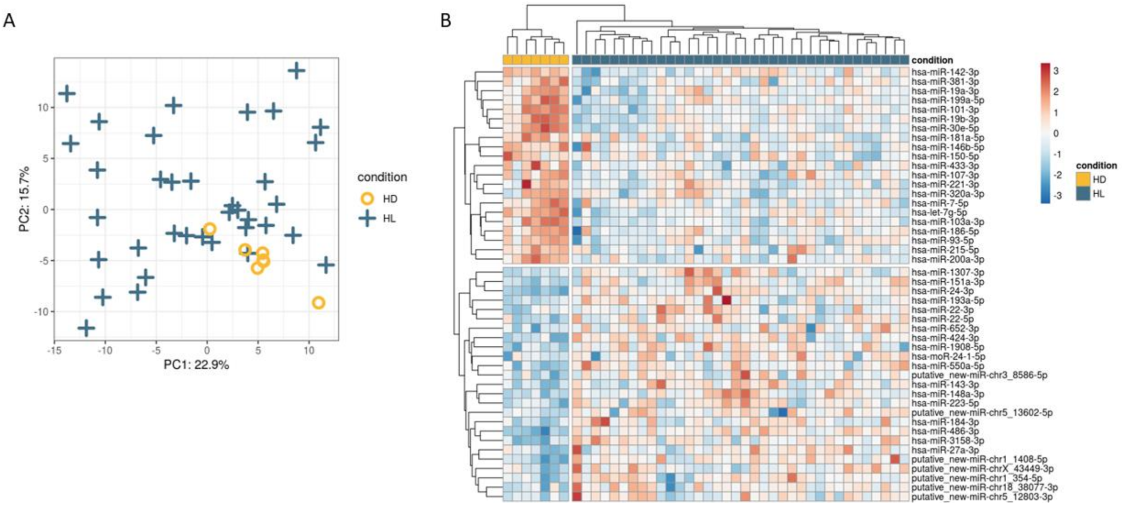

Figure S1. Small RNA (sRNA) sequencing of plasma circulating small extracellular vesicles derived RNA in 36 pediatric classical Hodgkin Lymphoma (cHL) cases and 7 healthy donors (HDs). (**A**) Principal component analysis of sRNA sequencing reveals a heterogeneous profile of cHL and similarity with HDs. (**B**) In cHL, 21 microRNAs (miRNAs) are less and 25 miRNAs/new putative miRNAs are more abundant compared to HDs.

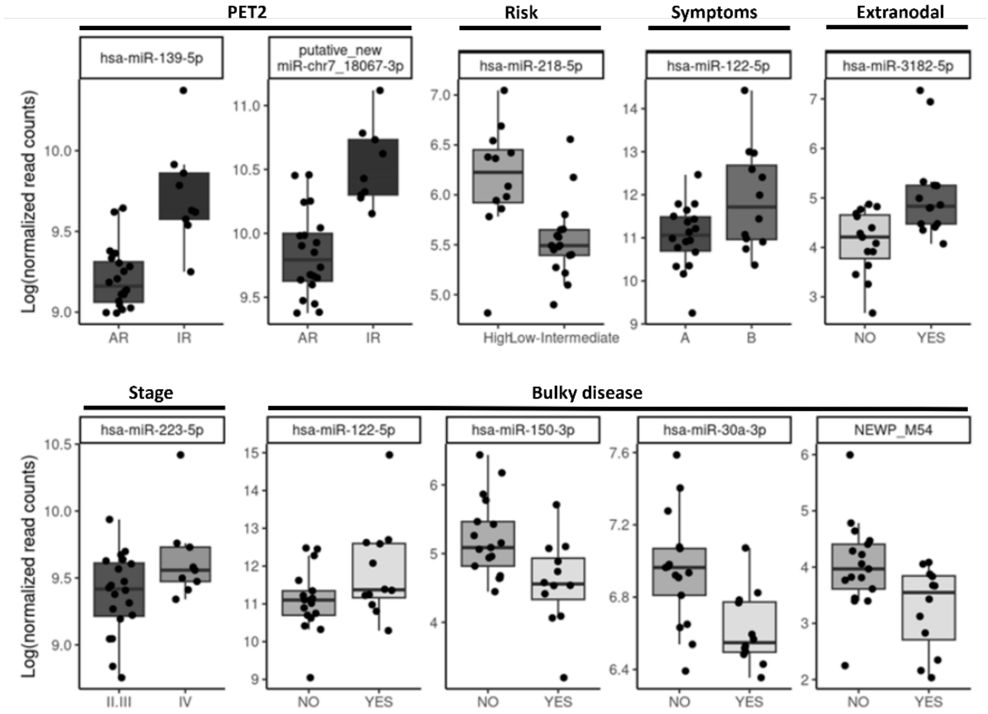

Figure S2. Integration of sRNA sequencing analysis with clinical data. Boxplots showing the differentially abundant microRNAs (miRNAs) in plasma sEVs samples from sRNA sequencing data in PET 2; High and low intermediate risk; patients presenting and not presenting B-symptoms, nodal vs. extranodal involvement, patients staged at diagnosis as stage IV vs. stages II and III; (**F**) bulky vs. non-bulky disease.

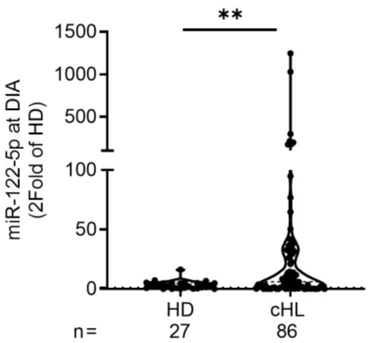

Figure S3. RT-qPCR analysis of miR-122-5p in 86 pediatric cHL plasma samples, 27 pediatric healthy donor (HD) plasma samples. At day of diagnosis (DIA) miR-122-5p expression is significantly different in plasma small extracellular vesicles of cHL patients compared to HDs. Mann–Whitney analysis of cHL vs. HDs: significant (*p*-value: 0.0098, **).

Table S1. The body mass index (BMI) and 2-fold of miR-122-5p at day of diagnosis (DIA) as a fold of healthy donors (HDs) positively correlate (*p*-value: 0.0033, **). Plasma small extracellular vesicle miR-122-5p levels were measured at DIA and follow-up (FUP) in 43 patients. The 2-fold at FUP is significantly increased compared to FUP. Data were analyzed using a Mann–Whitney test (*p*-value: 0.001, ****).

	**BMI vs. DIA miR-122-5p (2-fold of HDs)**	**DIA miR-122-5p (2-fold of HD) vs. FUP miR-122-5p (2-fold of HD)**
Statistical test	Spearman r	Mann–Whitney
Number of pairs	62	43
p-value	0.0033 (**)	<0.0001 (****)
